# Spectral Sensitivities and Color Signals in a Polymorphic Damselfly

**DOI:** 10.1371/journal.pone.0087972

**Published:** 2014-01-31

**Authors:** Shao-chang Huang, Tsyr-huei Chiou, Justin Marshall, Judith Reinhard

**Affiliations:** 1 Queensland Brain Institute, The University of Queensland, St Lucia, Queensland, Australia; 2 Department of Life Sciences, National Cheng Kung University, Tainan, Taiwan; National Tsing Hua University, Taiwan

## Abstract

Animal communication relies on conspicuous signals and compatible signal perception abilities. Good signal perception abilities are particularly important for polymorphic animals where mate choice can be a challenge. Behavioral studies suggest that polymorphic damselflies use their varying body colorations and/or color patterns as communication signal for mate choice and to control mating frequencies. However, solid evidence for this hypothesis combining physiological with spectral and behavioral data is scarce. We investigated this question in the Australian common blue tail damselfly, *Ischnura heterosticta*, which has pronounced female-limited polymorphism: andromorphs have a male-like blue coloration and gynomorphs display green/grey colors. We measured body color reflectance and investigated the visual capacities of each morph, showing that *I. heterosticta* have at least three types of photoreceptors sensitive to UV, blue, and green wavelength, and that this visual perception ability enables them to detect the spectral properties of the color signals emitted from the various color morphs in both males and females. We further demonstrate that different color morphs can be discriminated against each other and the vegetation based on color contrast. Finally, these findings were supported by field observations of natural mating pairs showing that mating partners are indeed chosen based on their body coloration. Our study provides the first comprehensive evidence for the function of body coloration on mate choice in polymorphic damselflies.

## Introduction

Signal emission and perception in a given visual system plays a crucial role in animal communication. Successful signaling via colors relies on the capacity of receivers to discriminate the target against the background noise [1], [2]. Good signal perception ability is particularly important for polymorphic animals, because the existence of multiple inter- and/or intra-sexual morphs is a significant challenge during mate choice in a reproductive site [3], [4]. The ability to accurately detect visual signals not only helps animals to recognize potential mates, it also reduces the energy cost involved in mate searching.

Ischnuran damselflies (Odonata: Zygoptera: Coenagrionidae) are an excellent animal model to investigate the evolutionary arms race between color signals and perception for two reasons. Firstly, ischnuran damselflies display diverse body colorations with polymorphism commonly confined to females, making mate choice a challenge [2], [5], [6]. Andromorph females have a male-like appearance, and gynomorph females express a distinctly different color to males or andromorphs. Female-limited polymorphism is believed to have evolved due to sexual conflicts over mating frequencies in terms of sexual selection [7]. Body coloration has always been assumed to be an important cue in mate choice and reproduction of ischnuran damselflies. However, to date there have been few detailed studies examining the spectral sensitivities of the visual photoreceptors and the body reflection in damselflies [2], [6], [8]. Whether and how the body colors indeed influence mate choice behavior needs to be investigated from the damselflies' visual perspective.

The second reason why ischnuran damselflies are a useful model for signaling and perception studies, lies in the fact that the Odonata as a whole (damselflies and dragonflies) have well-developed vision. Some have up to five photoreceptor types ranging from UV to long wavelengths [9]–[12]. Previous studies have interpreted the effect of multiple female morphs in damselflies on mating frequencies mainly from the human visual perspective [5], [13], [14], not taking into account that the perceptual capacity for colors significantly differs between humans and damselflies. Species-specific differences in visual spectral sensitivities can lead to a significant bias when analyzing color discrimination abilities.

Here, we used the Australian damselfly, *Ischnura heterosticta* to investigate whether and how their body coloration (signal emission) is correlated to their spectral discrimination ability (signal perception) in the context of mate choice. Similar to other ischnuran damselflies, *I. heterosticta* show female-limited polymorphism: andromorph females display a blue color conspicuous to the human eye, which is the same blue coloration that males display [15]. Gynomorph females, on the other hand, have distinctly different colorations including green, grey and intermediate forms [15]. *I. heterosticta* is unique in that this female polymorphism is split between immature and mature stages. Gynomorphs of *I. heterosticta* are mature, and andromorphs are immature females. After a period of 4–7 days, during which the andromorphs mature, an ontogenetic color change from male-like blue to gynomorphic green-grey occurs to signal sexual maturity and readiness for mating to males [16]. Mating in *I. heterosticta* occurs in the early morning just after sunrise [15]. Field observations and behavioral experiments suggest that the body coloration in *I. heterosticta* is an important cue for mate choice and sexual selection, as males predominantly prefer to mate with gynomorphic females and avoid the immature andromorphs [15], [16]. Although behavioral data indicate that color signals play a crucial role in reproduction of this species, physiological evidence regarding their spectral sensitivities is still lacking.

The aim of this study was to determine whether the color signals emitted by *I. heterosticta* morphs can be discriminated by their visual system, and whether males indeed use these signals for mate recognition when encountering polymorphic females. First we measured the spectral reflectance of the different *I. heterosticta* morphs, secondly we determined their photoreceptor spectral sensitivities using electrophysiological methods, and thirdly calculated chromatic and achromatic contrasts between individual morphs and the background from the visual perspective of *I. heterosticta*. Lastly, field observations on mating partner preferences were used for behavioral validation of the physiological prediction.

## Materials and Methods

### Animal collection

Damselflies were collected throughout the year from a pond in Fig Tree Pocket near Brisbane, Australia, and the lake on the St. Lucia campus of The University of Queensland, Australia. Damselflies were kept individually in a dark container with moist filter paper for no longer than two days before electrophysiology was conducted. Individuals were first used for electrophysiological recordings, and subsequently for color reflectance measurements. No permission is required for research on *I. heterosticta* in Australia as they are not protected or endangered species, and the studies were conducted in residential parks.

### Electroretinogram recordings

Electroretinography (ERG) is a quick method to examine the response from the overall visual system. Damselflies (12 males, 10 andromorphs, 11 gynomorphs) were cooled and dark-adapted for at least one hour before ERG recordings. Two chlorinated silver wires were attached to the corneal surface of the eyes with conductive gel serving as recording electrodes. Only one eye received a light stimulus, and the visual evoked potential differences between two corneal surfaces were amplified and recorded. Monochromatic light stimuli (50 ms) were presented in 10 nm steps from 300 to 700 nm and reverse. Three ERG measurements per individual were recorded; the results were averaged and pooled for each morph. For detailed methods here and below, see electronic Methods S1.

### Intracellular recordings

Intracellular recordings were conducted to determine the spectral sensitivity of individual photoreceptors of *I. heterosticta*. A sharp glass capillary filled with 1 M KCl solution was inserted into the eye to record the membrane potential of individual photoreceptor cells after stimulation with monochromatic light from 300 to 700 nm wavelength. A flash method [21] was applied to obtain spectral sensitivity curves of the photoreceptors, while the response-log stimulus intensity (*V/*log*I*) curve was also acquired for results correction. The spectral sensitivities of the examined photoreceptors were compared to a previously described template for visual pigments [17]. In total, 61 individuals (31 males, 12 andromorphs, 19 gynomorphs) were subjected to intracellular recordings, which included 7 UV cells, 17 Blue cells, and 52 Green cells.

### Spectral reflectance and irradiance measurements

The reflectance spectra of the bodies of the various damselfly morphs were acquired with a miniature spectrometer (USB-4000-UV-VIS, Ocean Optics, Inc., Dunedin, FL). In total, 31 males, and 103 females (19 andromorphs; 22 green, 47 intermediate, and 15 grey gynomorphs) were measured. Mixed plant leaves (n = 40) collected from the study site were measured as the background spectrum representing the locations where damselflies perched on vegetation. Two types of environmental light irradiance from the study sites, twilight (05:00), and morning light (06:00–08:00), were measured over a period of several days. Irradiance measurements were conducted hourly and the results were averaged for analyses.

### Calculation of chromatic/achromatic contrasts and discrimination values

By using the spectral sensitivities of the photoreceptors and the reflectance spectrum of each morph as well as the spectrum of the green background vegetation under different light irradiances, we calculated the receptor-specific chromatic and achromatic contrasts [18]–[20]. The receptor quantum catches (*Q_i_*) were calculated, and the receptor-specific contrast (*q_i_*), which is the quantum catch of each receptor class adapted to its light background was established. Discrimination values (Δ*S*) of the trichromatic visual system were calculated, with the units of Δ*S* being *jnd* (just noticeable differences). Achromatic contrast (brightness contrast) was also analysed by using the green sensitive receptor [22], [23]. An arbitrary *jnd* value of 1 was set as discrimination threshold in chromatic and achromatic contrasts based on a study using honeybees (*Apis mellifera*) [19] due to the lack of electrophysiological and behavioral evidence for damselflies.

We have applied ANOVA with Bonferroni cluster to compare chromatic as well as achromatic contrasts between inter-sexual color spectra, intra-sexual color spectra, and color spectra of morphs and vegetation under two light irradiance conditions (twilight and morning light) to determine the statistical difference with respect to the damselfly's visual system when viewing individual color morphs. One-tailed t-tests were applied to investigate whether both chromatic and achromatic contrasts were significantly different compared to the *jnd* threshold value 1, under both light irradiance conditions.

### Observation of morph frequencies from mating pairs

The observations of mating morph frequencies were conducted from 5:00 to 9:00 AM in December 2011 and January 2012 (n = 31 days). Comparisons of female morph numbers between mating pairs and the general female population were conducted to determine female morph preferences of males for mating. Results were analysed by G-test to determine male mate preference.

## Results

### Spectral sensitivities and responses of photoreceptor cells

**Figure 1:**
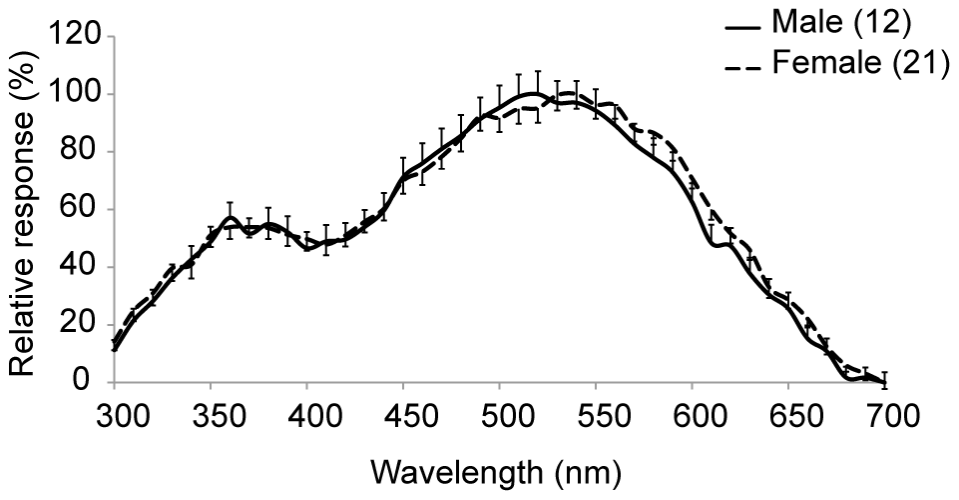
Electroretinogram (ERG) measurements from eyes of males and females of *Ischnura heterosticta*. Results are mean ± s.e. of males (solid line, 12 individuals) and females (dotted line, 10 andromorphs and 11 gynomorphs pooled together). Bars of s.e. are upward for males and downward for females.

**Figure 2:**
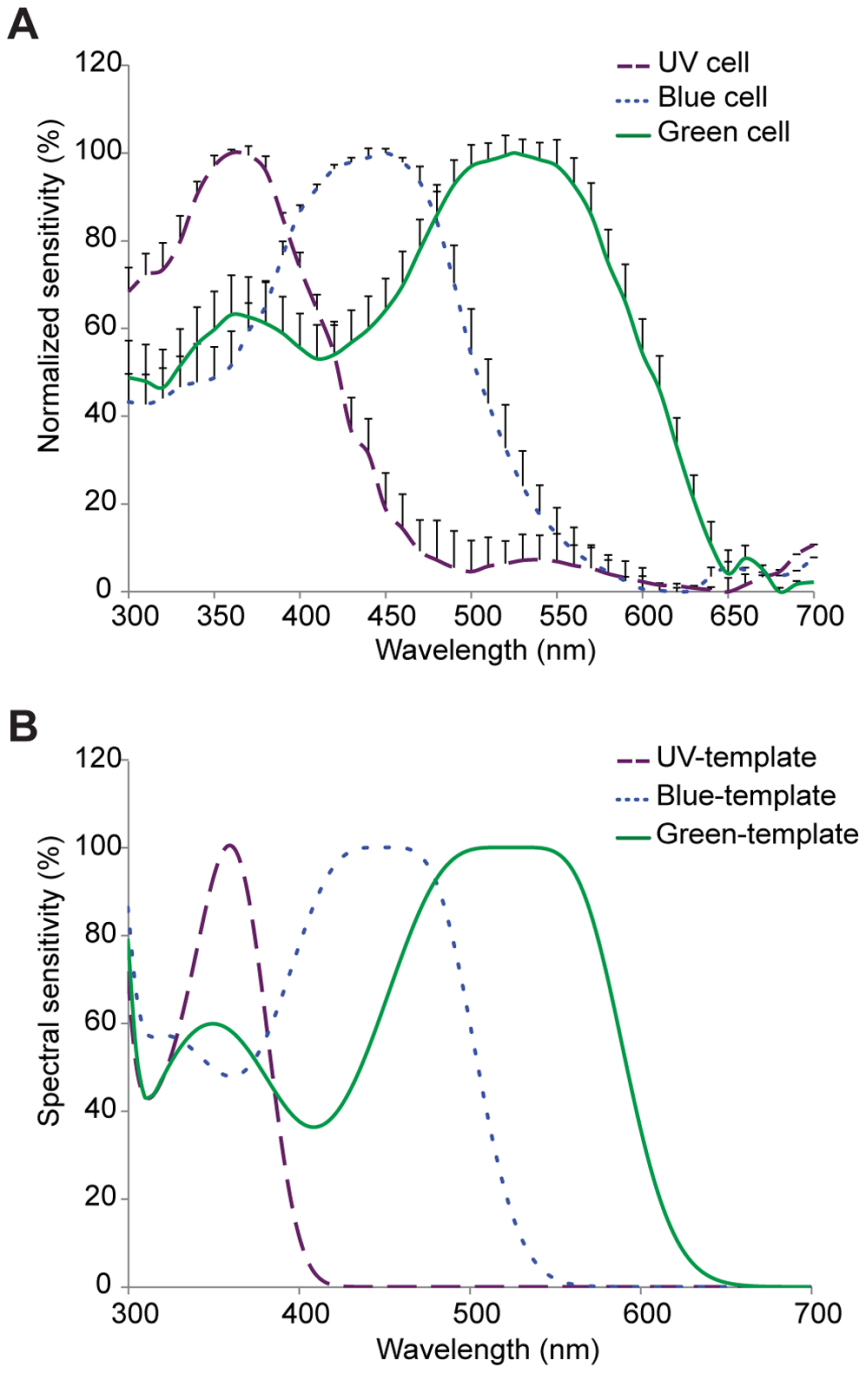
Spectral sensitivity of photoreceptor cells from intracellular recordings from the eyes of *Ischnura heterosticta*. (A) Spectral sensitivity curves (mean ± s.e.) established by calculating photon absorption of the examined photoreceptors using the intracellular recordings, with results normalized to 100% according to the maximum value of each photoreceptor; (B) curves generated from a template for visual pigments developed by Stavenga et al. [17] with λ_max_ at 360, 450, and 525 nm. Template curves (λ_max_ = 450, 525 nm) were adjusted for self screening based on Lambert-Beer's Law. Measurements were pooled from 61 individuals (31 males, 12 andromorphs, and 19 gynomorphs).

*I. heterosticta* showed no signs of sexual dimorphism in ERG recordings (Fig. 1). They were mostly sensitive to green light, with a primary peak at 520–540 nm, and a secondary peak in the ultraviolet (360 nm) (Fig. 1). Three types of photoreceptor cells (UV, blue, and green) were discovered using intracellular recordings. No sex differences were identified, and the data were thus pooled for all individuals (Fig. 2A). Three types of visual pigments, with maximal sensitivities of UV, blue and green receptors at λ_max_ = 360, 450 and 525 nm, respectively, were clearly distinguished from one another using the template derived from Stavenga et al. [17] (Fig. 2B).

### Color reflectance of *I. heterosticta*, vegetation, and light environment irradiance

**Figure 3:**
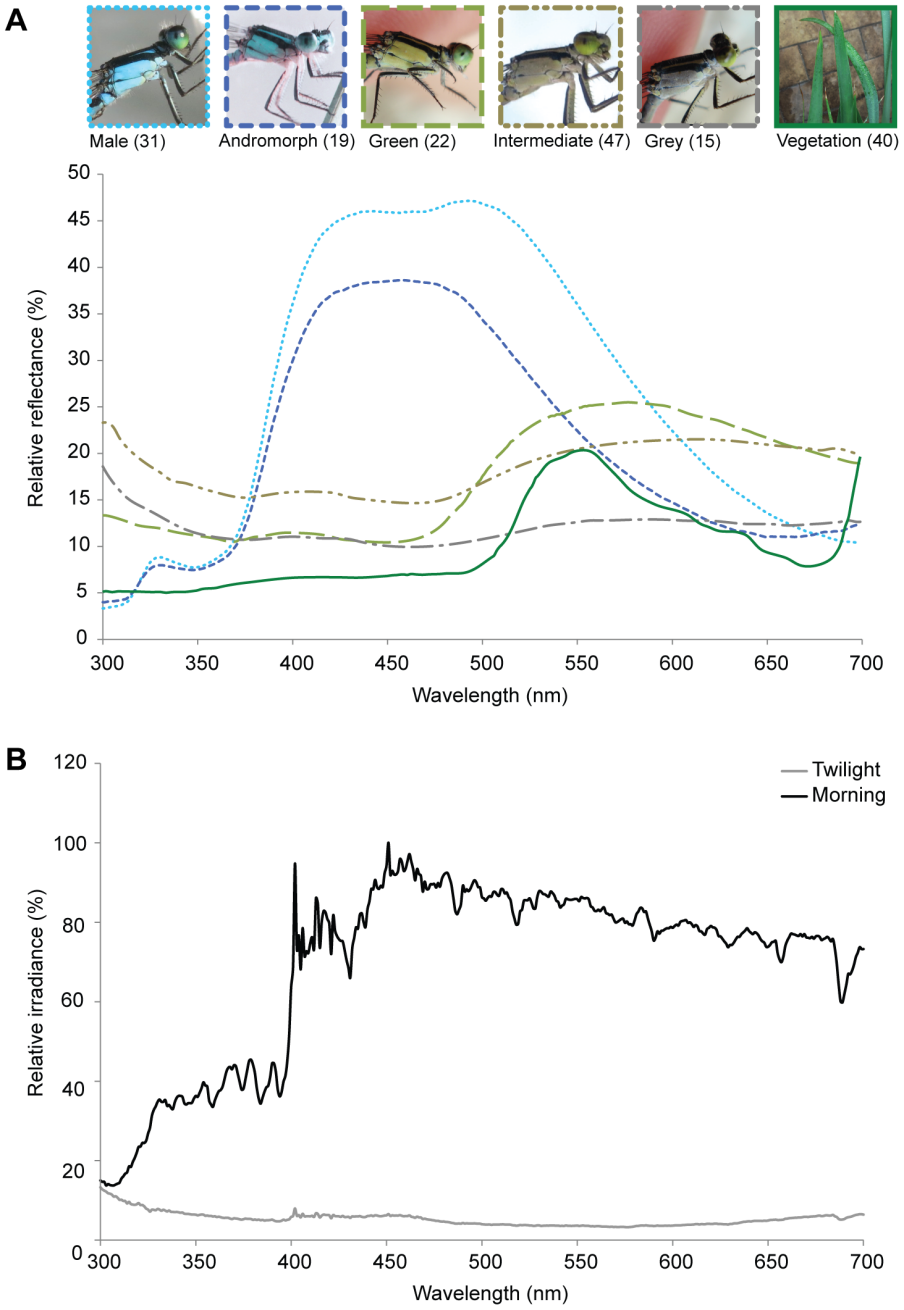
Relative spectral reflectances and irradiance. (A) Relative spectral reflectances from the bodies of males, andromorphs, and the three variants of gynomorphs of *Ischnura heterosticta*, and the green vegetation background (see Table S1 for descriptive statistics). Numbers in brackets represent sample sizes. (B) Relative irradiance of the ambient light environments from twilight and morning (mean) at the study sites (maximum photon numbers of twilight and morning are 1.16×10^19^ and 8.79×10^19^/s/cm^2^ respectively).

The bodies of males and andromorphs had similar reflectance spectra, with a major peak from 400 to 520 nm, accounting for their blue appearance, with the amplitude being slightly greater in males than andromorphs (Fig. 3A, Table S1). Green females had a peak between 520 and 620 nm, and reflectance below 470 nm remained low at ca. 10%. Grey females had a low reflectance spectrum along the entire wavelength range from 300 to 700 nm accounting for their dull grey coloration. Intermediate females had a constant reflectance across the entire measured wavelength range, similar to grey individuals but the amplitude was larger (Fig. 3A, Table S1). The reflectance spectra collected from vegetation showed a peak between 530 to 560 nm (Fig. 3A, Table S1). Environmental irradiance measurements revealed that light intensity, i.e. the relative photon number compared to the maximum, was much lower under twilight than under morning conditions (Fig. 3B).

### Discrimination capacity based on chromatic and achromatic contrasts

**Figure 4:**
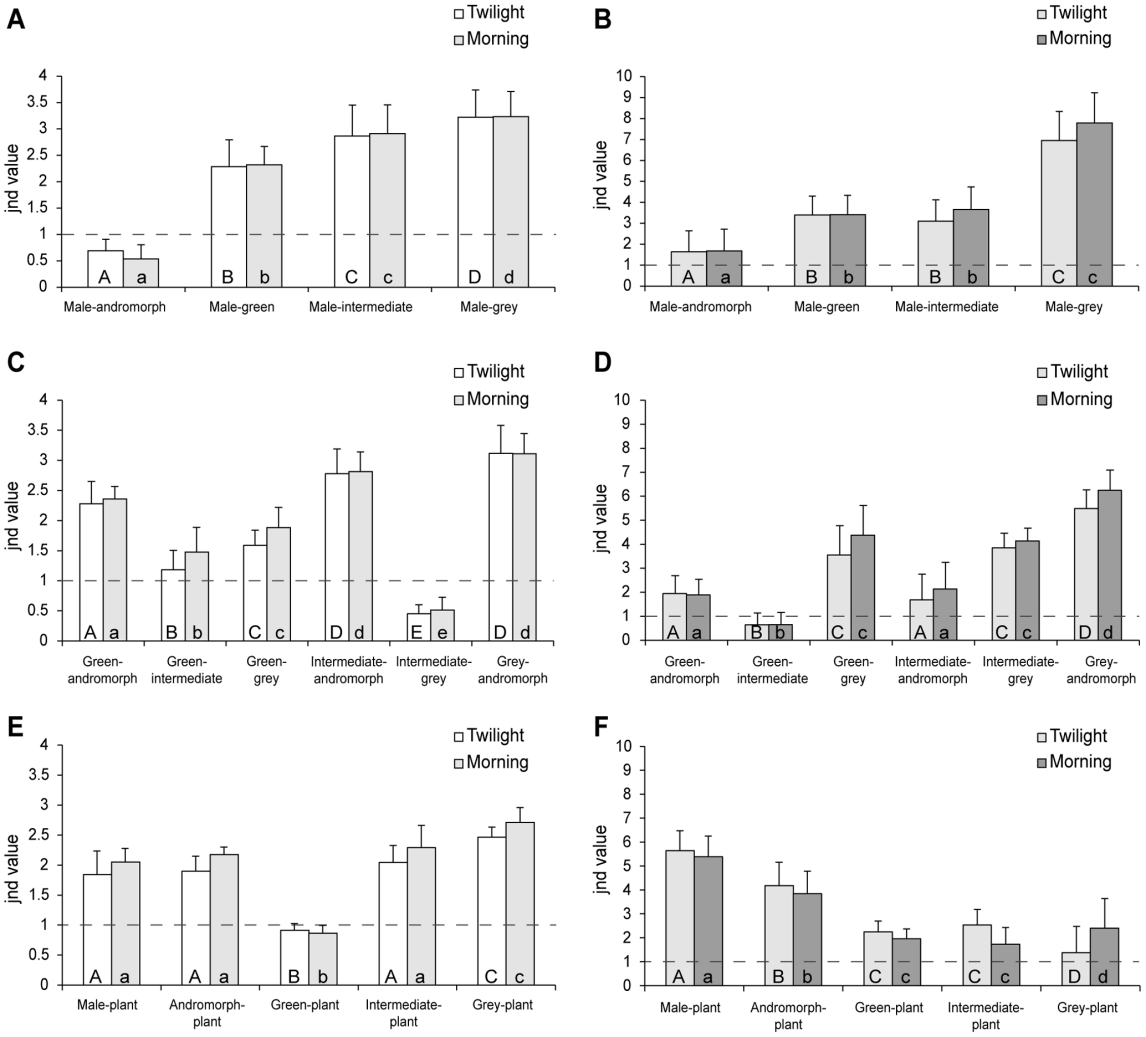
Chromatic and achromatic contrasts. Pair-wise comparisons in *Ischnura heterosticta* under twilight and morning irradiance (mean ± s.d.). *Jnd* value: ‘just noticeable differences’ used as discrimination index; a *jnd* value of 1 has been set as discrimination threshold value (dashed line) (A) Chromatic and (B) achromatic contrast comparisons between males and females; (C) chromatic and (D) achromatic contrast comparisons between female morphs; (E) chromatic and (F) achromatic contrast comparisons between individuals and vegetation. ANOVA with Bonferroni cluster was used to analyse overall differences within each group separately for twilight and morning light conditions (see text for statistical results), and different letters inside bars signify statistical difference at *P*<0.001. For statistical results of the comparisons of contrasts to the *jnd* threshold value 1, see Table S2.

The chromatic contrast calculations showed that males and andromorphs (*jnd*: twilight = 0.69; morning light = 0.53) appear similar to the visual system of *I. heterosticta* (*jnd*<1) compared to other inter-sexual comparisons under both twilight and morning light environments (Fig. 4A, twilight: *F_3, 3194_* = 787.26, *p*<0.01; morning light: *F_3, 3194_* = 1630.47, *p*<0.01; see also Table S2). That is, males and andromorphs are most likely difficult to discriminate for *I. heterosticta* due to their similar color reflectance. In contrast, the three types of gynomorphs can be clearly distinguished from males (*jnd* values range between 2.28 and 3.23 under twilight and morning light, Fig. 4A, Table S2), providing the evidence that *I. heterosticta* is able to discriminate gynomorphs from males based on the chromatic contrast between them (Table S2). Unlike the chromatic results, achromatic contrasts between males and females all showed a higher *jnd* value (ranging from 1.63 to 7.79, Fig. 4B) suggesting that *I. heterosticta* could utilize achromatic signals to distinguish different genders and morphs (Fig. 4B, twilight: *F_3, 3194_* = 711.30, *p*<0.01; morning: *F_3, 3194_* = 864.74, *p*<0.01; Table S2).

A high *jnd* value in intra-female pair-wise comparisons (ranging from 1.18 to 3.11, Fig. 4C) suggested that all could be visually discriminated based on chromatic contrast alone under both light conditions except for intermediate vs grey females (Fig. 4C, twilight: *jnd* = 0.45, *t_704_* = 19.56, *p*<0.01; morning: *jnd* = 0.51, *t_704_* = 24.45, *p*<0.01, details see Table S2). Achromatic comparisons between any two female morphs all showed high contrast (*jnd* values ranging from 1.68 to 6.25, Fig. 4D) except for the comparison between green and intermediate morphs (Fig. 4D, twilight: *jnd* = 0.64, *t_1033_ = 215.4*, *p*<0.01; morning: *jnd* = 0.50, *t_1033_ = 228.4*, *p*<0.01, details see Table S2).

The comparisons between individuals and vegetation showed significantly lower discrimination values between green females and vegetation (*jnd* values: twilight = 0.91, morning = 0.86, Fig. 4E, Table S2) than for the other morphs (*jnd* values ranging from 1.84 to 2.70, Fig. 4E, Table S2) suggesting that green females camouflage against the surrounding vegetation efficiently, while the other morphs were conspicuous against green vegetation (Fig. 4E, twilight: *F_4,5359_* = 568.82, *p*<0.01; morning: *F_4,5359_* = 842.24, *p*<0.01). Achromatic contrast suggests that the intensity signals from the various morphs could be discriminated against the background by the *I. heterosticta* visual system, including the green females (*jnd* values ranging from 1.37 to 5.64, Fig. 4F, twilight: *F_4,5359_* = 687.96, *p*<0.01; morning: *F_4,5359_* = 548.52, *p*<0.01, for details see Table S2).

### Mate choice preference

**Figure 5:**
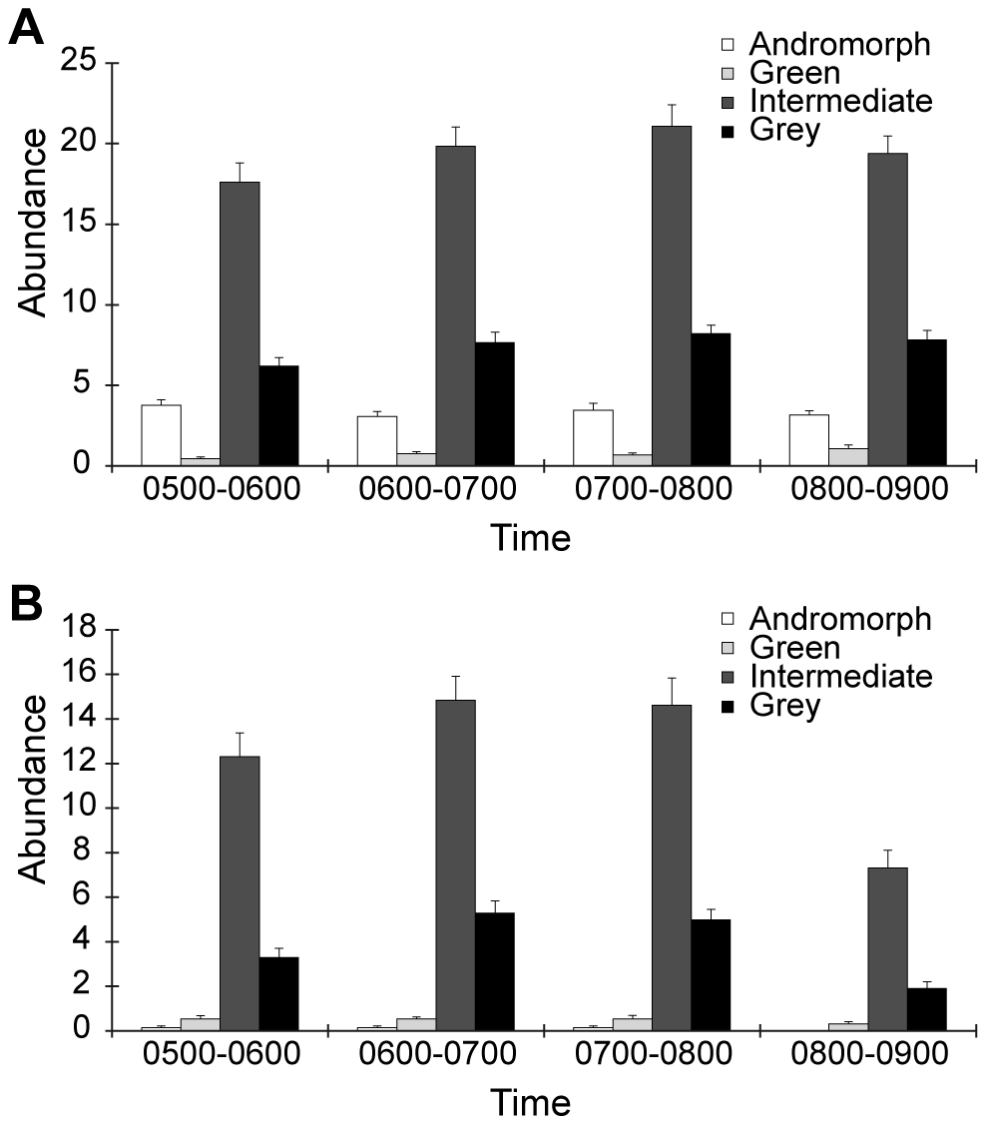
Female morph frequencies in the damselfly *Ischnura heterosticta*. Female morph frequencies were recorded from (A) the overall population, and (B) mating pairs during the first four hours after sunrise. Shown are means ± s.d., n = 31 days. Statistical comparison between (A) and (B) described in the text.

Results of mate choice observations from 869 mating pairs from the field showed that males preferred predominantly to mate with gynomorphs (>99%) and ignored andromorphs, although they comprised 11% of the female population in the field (Fig. 5, G_0.05,3_ = 33.97, *p*<0.01 for 5–6 AM; G_0.05,3_ = 25.56, *p*<0.01 for 6–7 AM; G_0.05,3_ = 27.27, *p*<0.01 for 7–8 AM, and G_0.05,3_ = 18.11, *p*<0.05 for 8–9 AM). Over the entire observation period, only two andromorphs were observed mating with males, which suggested that males did not recognize andromorphs as mating partners.

## Discussion

The vibrant body coloration has always been considered an important cue for Odonata to recognize and assess mate quality [24], [25], but solid evidence combining physiological with spectral and behavioral data is scarce. Here, we provide first evidence that ischnuran damselflies of the species *Ischnura heterosticta* have three spectral classes of photoreceptors, which suggests the potential of color vision. We further show that their visual system can detect the spectral properties of the various color morphs in both males and females, and that the morphs can be discriminated against each other and the vegetation based on theoretical contrast calculations. Finally, these findings were supported by field observations of natural mating pairs showing that mating partners are indeed chosen based on their body coloration.

### Spectral sensitivities and color signals

Well-developed visual systems are found in many Odonata [9]–[12]. Our study confirms this in demonstrating that *I. heterosticta* also have three spectral photoreceptor types, being able to detect UV, blue, and green wavelengths of light. It differs, however, from the vision of other dragonfly and damselfly species (e.g. *Sympetrum rubicundulum*, *Hemicordulia tau*, and *I. elegans*), that may have four or five photoreceptor types sensitive from UV to long wavelength light, and that also display various body colors potentially used for mate discrimination [8], [11], [12]. Although our electrophysiological data cannot entirely rule out the possibility of tetrachromatic color vision also in *I. heterosticta*, trichromacy seems more likely. Evidence at the molecular level shows that *Telebasis salva* (Coenagrionidae) damselflies have only three copies of opsin genes, namely UV, blue and LW opsins [26], and preliminary results from our laboratory similarly identified three opsin genes in *I. heterosticta* (Huang et al., unpublished data). This is consistent with the idea that Odonata are ancient insects whose visual system originates from UV-blue-green-trichromacy of the ancestor of all pterygote insects [9]. The tetrachromacy found in a closely related species, *I. elegans* [8] may suggest that the two types of LW photoreceptors could have risen from gene duplication; however there are no studies to date that could confirm this.

### Use of color signals for mate recognition

Visual signals are widely used in Odonata to recognize conspecifics, and include body shape [27], wing coloration [28], [29], and body coloration [2], [6], [24], [30], [31]. Artificial modification of the natural body coloration of coenagrionid damselflies showed that color signals might be important for mate recognition [24]. Our study confirms this by providing solid physiological evidence of three photoreceptor types in *I. heterosticta* as basis of color vision, that enables them to discriminate relevant spectral differences, and by calculating the chromatic and achromatic contrasts between the various morphs. The latter showed that *I. heterosticta* vision is equipped to detect the color difference between gynomorphs and andromorphs, but has difficulties distinguishing between males and andromorphs. This makes sense in light of the fact that andromorphs are immature and their blue body color may be a signal to avoid mating [16]. Furthermore, new results of male mate choice experiments that manipulated female body colorations confirmed that *I. heterosticta* have color vision and indeed use it for mate selection (Huang and Reinhard, unpublished data). Thus, our results demonstrate that this species is physiologically able to use body color signals to correctly detect, identify and discriminate mating partners.

Van Gossum et al. [6], and Schultz et al. [2] used a similar method to show the importance of color signals for discrimination of individual morphs. However, unlike ours neither of the above studies included spectral sensitivity data from a specific damselfly species for their calculations, which can significantly bias the calculated color and contrast discrimination abilities. Also, our field observations provide additional behavioral evidence that *I. heterosticta* choose mating partners based on their body colors, as males predominantly mate with gynomorphs and mostly ignore andromorphs, although the latter comprise nearly 11% of the female population. Clearly, males recognize the grey-green gynomorphs as the mature females, which are ready for mating, and avoid the blue andromorphs, whose body color signals sexual immaturity [16].

### Mating with andromorphs: a case of mistaken mate recognition?

Although *I. heterosticta* males generally avoid mating with the immature blue andromorphs, we still observed males mating with andromorphs on rare occasions. Have males in this case made “a visual mistake” and incorrectly chosen an andromorph, or have they “deliberately” chosen an andromorph? Although andromorphs are very similar to males, their color reflectance is less intense and under certain conditions andromorphs may be distinguished as females and chosen as mates. In a number of *Ischnura* species, male damselflies mate with andromorphs: for example, when the proportion of andromorphs suddenly increases [25], [32]–[34], or when andromorphs become the preferred choice due to a previous mating experience with an andromorph [30], [35]. In case of *I. heterosticta*, however, neither scenario is likely. As all andromorphs eventually change color to gynomorphs [16], they can never achieve or maintain a high enough proportion within a population, which could lead to more regular mate choice of andromorphs. Furthermore, with andromorphs not predominant in the field, and mostly being considered rivals by males and being aggressively harassed [16], mating with andromorphs is so rare that it prevents mate choice learning.

The above makes it unlikely that on the three observed occasions males have “deliberately” chosen an *I. heterosticta* andromorph for mating. Have they made “a visual mistake”? The body color spectra and chromatic contrast calculations show that males can clearly distinguish andromorphs from gynomorphs based on color. However, under low light conditions color signals are variable and many insects rely on achromatic contrast information rather than color to distinguish targets from a distance [22], [23], as well as for motion detection, including mate or prey detection [36]. Considering the higher just noticeable difference (*jnd*) values of achromatic contrast among different morphs and that *I. heterosticta* starts to form mating pairs during twilight, it is conceivable that under the low light condition males of *I. heterosticta* use achromatic contrast information rather than color to detect females, and may occasionally confuse an andromorph for an intermediate female.

### Detection of color signals against vegetation

Effective signal detection against background noise plays an important role in successful animal communication [37], [38]. *Ischnura heterosticta* are commonly found perching on green vegetation, and accurate mate detection and recognition thus depends on how the color signals emitted by the different morphs contrast against vegetation. We calculated this contrast using the actual spectral discrimination ability of *I. heterosticta*, to determine whether they could theoretically detect any of their own morphs against a background of plants. The results show that for *I. heterosticta* eyes each morph is significantly distinguishable from the vegetation background including the grey and intermediate females. The only exception is the green gynomorphs, which have chromatically a very similar reflectance spectrum to the vegetation. The fact that green gynomorphs are hard to distinguish from a plant background, explains the less frequent mating occurrences for green gynomorphs compared to grey and intermediate gynomorphs, as males have difficulties detecting the green individuals on vegetation. Individual color variation in Odonata has been suggested to be an indicator for health and energy status [28], [29]. Green females of *I. heterosticta* are thought to be young adults with fewer energy reserves [15], and mating might therefore not be the first priority for them. They can modify their body color to grey or intermediate [16] later on, which increases their mating frequencies.

### Evolution of female color polymorphism

Intraspecific mate recognition is a challenge for male ischnuran damselflies as polymorphic females exist sympatrically, and one might argue that multiple photoreceptors in *Ischnura* have evolved to cope with this challenge – or on the other hand that the evolution of diverse spectral sensitivities has driven female polymorphism in *Ischnura*. Benefits and costs of females being polymorphic has been the matter of much debate, as a cost-benefit balance would explain why female-limited polymorphism exists and why it is maintained [5], [39]. In *I. heterosticta*, andromorphs are a unique case, as they are all immature individuals. They maintain their blue coloration during immaturity to mimic males, which provides a protection against excessive long-term mating duration [16]. Once they reach maturity, andromorphs change their body coloration into gynomorph resulting in successful mating and oviposition [16]. Our present study showed that based on reflectance spectra and spectral sensitivity *I. heterosticta* males cannot recognize andromorphs as females and thus generally do not attempt to mate with them. While this is of benefit to the andromorphs during immaturity, eventually these individuals mature and need to mate – which they cannot achieve if they would remain blue. Hence, andromorphs have to change color from male-like blue into gynomorphic green-grey to be recognized as mature females ready to mate. Whether the existence of three spectral photoreceptors in *I. heterosticta* is the driving force for evolution and maintenance of female polymorphism in this species, and for the ontogenetic color change in andromorphs remains to be investigated.

## Supporting Information

Methods S1 **Full methods.**
(PDF)

Table S1 **Descriptive statistical values of the colour reflectance of each morph.**
(PDF)

Table S2 **One sample t-test of chromatic and achromatic contrasts under twilight and morning light compared with the** ***jnd*** **threshold value, 1.**
(PDF)
