# Supplementary material for: Spectral Sensitivities and Color Signals in a Polymorphic Damselfly
Source: PLoS One. 2014 Jan 31;9(1):e87972. doi: 10.1371/journal.pone.0087972 (PMC3909319; doi:10.1371/journal.pone.0087972)
Supplement: Methods S1 — Full methods. (PDF) [file pone.0087972.s001.pdf]

Supporting Information: Full methods

Light stimuli for electrophysiology

A 150W Xenon arc lamp (Thermo Oriel, USA) was used as the light source for the electrophysiology. Monochromatic light was produced by a computer-controlled monochromator (model 7340, Oriel Instruments, USA) and delivered via a 520 μm optic fiber (model 77557, Newport, USA) 15 to 18 cm in front of the test animal's eyes. Stimulus brightness was controlled by a UV-grade circular linear variable neutral density filter (ND, 4 log units). During the test, light flow was calibrated by adjusting ND filters to produce equal quantum flux at each wavelength step of the measurement. A shutter (LS6, Uniblitz, USA) was used to control light duration of the flashes. Visual stimuli (50 ms) were presented with an interval of two seconds between measuring steps. To eliminate the potential effect of chromatic adaptation during the experiment, light stimuli from short to long wavelengths and the reverse direction were both recorded.

Electroretinogram recordings

Electroretinogram (ERG) is a quick method to examine the response from the overall visual system. Before ERG recordings, freshly collected individuals (12 males, 10 andromorphs, 11 gynomorphs) were kept in a fridge at 8°C for at least one hour, and dark-adapted for at least 30 minutes before ERG was conducted. Appendages of damselflies were removed and the openings were sealed with Vaseline to prevent the loss of body fluid. Damselflies were placed on a platform and immobilized with a mixture of beeswax and resin (3:1). The platform was then transferred into a Faraday cage with the eyes of the individual located at the center of the light stimulus apparatus and facing horizontally. Two chlorinated silver wires serving as recording electrodes were attached with conductive gel (Sigma gel, Parker laboratory Inc. USA) to the same region of cornea surface of the two compound eyes to compare evoked potential differences between two corneal surfaces after light stimulation of one eye. A third chlorinated silver wire was inserted into the thorax as the indifferent electrode. The recorded signal was pre-amplified with an AC differential amplifier (DP-301, Warner Instrument Corp., USA) with band-pass filters from 1 Hz to 1 kHz. The

amplified signal was acquired and transferred to a desktop PC for data analysis using a multifunction data acquisition device (NI USB-6009, National Instruments, USA). Each damselfly was tested within two hours after preparation and three ERG measurements were taken from each subject. The results were averaged and pooled for each morph.

Intracellular recordings

In order to determine the spectral sensitivity of individual photoreceptors in the eyes of *I. heterosticta*, intracellular recordings were conducted. The animal was mounted the same way as described for the ERG measurements. Using a razor blade, a small triangular hole (covering around 150 facets) was cut in the left eye under dim red light illumination. The hole was immediately sealed with a drop of silicone lubricant (Molytec, Australia) to prevent clotting and dehydration. The animal was positioned with an eye in the center of a rotatable optic fiber holder in the Faraday cage. A chlorinated silver wire was inserted into the thorax as an indifferent electrode and surrounded with conductive gel. Sharp glass capillaries (GC 120F-15, OD: 1.2 mm, ID: 0.69 mm, Harvard Apparatus Ltd., Kent, UK) were pulled using a micropipette puller (P-97; Sutter Instrument Co., Novato, California, USA), filled with 1 M KCl solution (yielding 80 to 160 MΩ resistance), mounted on an electrode holder head stage (HS-2, Axon Instruments, USA) and inserted into the eye through the opening.

The glass electrode was slowly lowered into the tissue using a water-filled hydraulic micromanipulator (MMW-20, Narishige Co. Ltd. Japan). Once the membrane potential dropped below -25 mV, a white light from an external strobe (Q15, Quantaray, Japan) was delivered to test if a depolarized response could be elicited as indication that the electrode had penetrated a photoreceptor. Further white light flashes were delivered from various angles to identify the optimal receptive field of the penetrated photoreceptor. The damselfly was then dark adapted for 30 minutes before measurement.

Response-log stimulus intensity (*V*/log*I*) curve was measured (17 white light intensities over 4 log units with 50 ms flash in 5 s interval). A flash method [1] was applied, and each photoreceptor cell was examined three times between 300 to 700 nm in both directions. The recorded signal was amplified by a multipurpose

microelectrode amplifier (AXOPROBE-1A, AXON Instruments, USA) and analysed as for the ERG. Spectral sensitivity curves of the photoreceptors were corrected based on the results of *V*/log*I*. A hyperbolic function:

$$V / V_{max} = (R*I)^n / \left((R*I)^n + 1\right) \quad (1),$$

where *I* is stimulus intensity in quantal flux; *V* is the amplitude of the receptor response in mV; *Vmax* is the saturated response amplitude; *R* is the intensity yielding a 50% response of *Vmax*; *n* is a constant determining the slope of the function (coefficient n values for UV cells: 0.657± 0.069; blue cells: 0.569± 0.072; green cells: 0.694± 0.142) (details see [1,2]). Photon absorption at different wavelengths of light was calculated accordingly. The spectral sensitivities of the examined photoreceptors were compared to a previously described template for visual pigments [3]. In total, 61 individuals (31 males, 12 andromorphs, 19 gynomorphs) were tested using intracellular recordings, which included 7 UV cells, 17 Blue cells, 52 Green cells.

Spectral reflectance and irradiance measurements

The reflectance spectra of the bodies of the various damselfly morphs were acquired with a miniature spectrometer (USB-4000-UV-VIS, Ocean Optics, Inc., Dunedin, FL). The samples were illuminated with a 150 W Xenon lamp (Thermo Oriel, USA). Damselflies from the electrophysiological experiments and field collections were placed on a horizontal platform and measurements were taken from thoraces through an optic-fiber cable (P100-2-UV-VIS, Ocean Optics, Dunedin, Florida, USA). In order to calculate the relative reflectance spectra of the sample, we also took measurements from a WS-1 diffuse reflectance standard (Ocean Optics) under the same conditions. We measured the spectral reflectance from 31 males, and 103 females (19 andromorphs, 22 green gynomorphs, 47 intermediate gynomorphs, and 15 grey gynomorphs). Three measurements per individual were taken on the thorax where blue, green, and grey colorations appeared. Reflectance results were averaged to 1 nm intervals ranging from 300 to 700 nm.

Spectral irradiance was measured using the same spectrometer (USB-4000, Ocean Optics), which was first calibrated using DH-2000 (Ocean Optics) as the standard light source to obtain the radiant energy conversion ratio for the

spectrometer. This allowed us to convert irradiance measurements from energy units into photons. In order to acquire the spectral irradiance of the vegetation background, mixed plant leaves (n= 40) collected from the study site were measured, and reflectance spectra were pooled as the background spectrum representing the locations where damselflies perched on vegetation. Lastly, we measured the ambient environmental light conditions, focusing on the morning hours when *I. heterosticta* predominantly mates. *I. heterosticta* start to form copulation pairs after sunrise, and the light intensity varies dramatically during the first few hours after dawn. We therefore measured two types of environmental light irradiance from the study site, twilight (05:00), and morning (06:00-08:00). Twilight irradiance was measured from 23rd to 26th October 2011. Morning irradiance was collected from 3rd to 7th October 2011. All measurements were conducted hourly and the results were averaged for analyses.

Calculation of chromatic/achromatic contrasts and discrimination values

By using the spectral sensitivities of the photoreceptors and the reflectance spectrum of each morph as well as the spectrum of the green background vegetation under different light irradiances, we calculated the receptor-specific chromatic and achromatic contrasts [4-6]. The receptor quantum catches (Q_i) were calculated as:

$$Q_i = \int_{300}^{700} S_i(\lambda) I(\lambda) R(\lambda) d\lambda \quad (2),$$

where i denotes the spectral types of receptor (UV, B, G), $S_i(\lambda)$ is the spectral sensitivity function of the receptor i, $I(\lambda)$ is the illumination spectrum, and $R(\lambda)$ is the reflectance spectrum of each morph of individuals or green vegetation. The receptor-specific contrast (q_i), which is the quantum catch of each receptor class adapted to its light background was established as,

$$q_i = Q_i / Q_i^B \quad (3),$$

where Q_i^B is the adaptation coefficient of a receptor to its light environment by using the von Kries transformation [7].

Discrimination values (ΔS) of the trichromatic visual system were calculated according to equation (3). Intensity (brightness) cues are ignored in this model.

$$(\Delta S)^2 = \frac{[\omega_1^2(\Delta f_3 - \Delta f_2)^2 + \omega_2^2(\Delta f_3 - \Delta f_1)^2 + \omega_3^2(\Delta f_1 - \Delta f_2)^2]}{[(\omega_1\omega_2)^2 + (\omega_1\omega_3)^2 + (\omega_2\omega_3)^2]} \quad (4),$$

where ω_i is the noise value of each receptor class (ω_{UV}= 0.207,ω_B= 0.244, ω_G= 0.217, values were adapted from equation (5) and (6) in Vorobyev et al. [5]. f_i= Ln(q_i) is the log transformed receptor-specific contrast and Δf_i is the difference in a receptor between two stimuli (pair-wise comparisons between different morphs or with the green vegetation). The units of ΔS are *jnd* (just noticeable differences).

Achromatic contrast (brightness contrast) was also analysed by using the green sensitive receptor (*i*) [8.9],

$$\Delta S = \left|\Delta f_i/\omega\right| \quad (5).$$

Electrophysiological and behavioral evidence to determine the definitive discriminated threshold value in chromatic and achromatic contrasts for damselflies was not available. Therefore we have set an arbitrary *jnd* value of 1 as discrimination threshold, based on a previous study using honeybees (*Apis mellifera*) [5]. We have applied ANOVA with Bonferroni cluster to compare chromatic as well as achromatic contrasts between inter-sexual color spectra, intra-sexual color spectra, and color spectra of morphs and vegetation under two light irradiance conditions (twilight and morning light) to determine the statistical difference with respect to the damselfly's visual system when viewing individual color morphs. One-tailed t-tests were applied to investigate whether both chromatic and achromatic contrasts were significantly different compared to the *jnd* threshold value 1, under both light irradiance conditions.

Observation of morph frequencies from mating pairs

The observations of mating morph frequencies were conducted from 5:00 to 9:00 AM between December 2011 and January 2012 (n= 31 days). Comparisons of female morph numbers between mating pairs and the general female population were conducted to determine female morph preferences of males for mating. Results were analysed by G-test to determine male mate preference.

References

1 Menzel R, Ventura DF, Hertel H, de Souza JM, Greggers U (1986) Spectral sensitivity of photoreceptors in insect compound eyes: comparison of species and methods. J Comp Physiol A 158: 165-177.

2 Matić T, Laughlin SB (1981) Changes in the intensity-response function of an insect's photoreceptors due to light adaptation. J Comp Physiol A 145: 169-177.

3 Stavenga DG, Smits RP, Hoenders BJ (1993) Simple exponential functions describing the absorbance bands of visual pigment spectra. Vision Res 33: 1011-1017.

4 Vorobyev M, Osorio D, Bennett ATD, Marshall NJ, Cuthill IC (1998) Tetrachromacy, oil droplets and bird plumage colours. J Comp Physiol A 183: 621-633.

5 Vorobyev M, Brandt R, Peitsch D, Laughlin SB, Menzel R (2001) Colour thresholds and receptor noise: behaviour and physiology compared. Vision Res 41: 639-653.

6 Vorobyev M, Osorio D (1998) Receptor noise as a determinant of colour thresholds. Proc R Soc Lond B 265: 351-358.

7 Foster DH, Nascimento SMC (1994) Relational colour constancy from invariant cone-excitation ratios. Proc R Soc Lond B 257: 115-121.

8 Spaethe J, Tautz J, Chittka L (2001) Visual constraints in foraging bumblebees: Flower size and color affect search time and flight behavior. Proc Natl Acad Sci U S A 98: 3898-3903.
