# Supplementary material for: Spectral Sensitivities and Color Signals in a Polymorphic Damselfly
Source: PLoS One. 2014 Jan 31;9(1):e87972. doi: 10.1371/journal.pone.0087972 (PMC3909319; doi:10.1371/journal.pone.0087972)
Supplement: Table S1 — Descriptive statistical values of the colour reflectance of each morph. (PDF) [file pone.0087972.s002.pdf]

Supporting Information

Table S1. Descriptive statistical values of the colour reflectance of each morph. Results are presented at 50nm interval between 300 to 700nm from 31 males, 19 andromorphs, 22 green gynomorphs, 47 intermediate gynomorphs, 15 grey gynomorphs, and 40 vegetation samples. S.D. is the standard deviation; min. is the minimum value; 25% is the first quartile (25th percentile); 50% is the median value (50th percentile); 75% is the third quartile (75th percentile); max. is the maximum value.

		300nm	350nm	400nm	450nm	500nm	550nm	600nm	650nm	700nm
Males	Mean	3.33	7.73	36.07	45.92	46.83	36.02	22.42	13.56	10.37
	S.D.	0.88	2.54	6.24	5.54	6.21	5.48	3.45	2.84	2.02
	Min.	1.12	4.54	21.85	29.61	31.66	29.31	16.54	5.24	4.84
	25%	1.84	5.12	32.52	39.83	39.59	33.62	18.24	11.21	7.15
	50%	3.25	7.84	39.87	46.65	46.65	39.99	21.95	13.89	10.03
	75%	5.24	10.54	43.01	48.44	50.37	44.56	25.21	15.28	12.89
	Max.	7.15	13.54	51.43	58.02	57.56	50.41	30.15	18.54	15.21
Andromorphs	Mean	4.01	7.51	29.94	38.51	34.36	22.42	14.72	11.01	12.41
	S.D.	1.02	2.85	4.84	6.12	5.21	3.12	4.84	4.18	2.54
	Min.	2.21	5.02	18.76	23.56	21.81	17.74	12.47	8.94	9.86
	25%	2.84	6.02	27.93	29.38	29.76	22.81	14.95	11.04	10.54
	50%	4.15	7.48	29.85	34.55	30.35	23.26	14.98	11.93	11.24
	75%	5.85	8.85	32.94	40.58	37.05	29.71	21.41	16.28	14.85
	Max.	6.02	11.51	39.89	49.25	45.99	32.54	33.38	26.24	20.84
Green	Mean	13.31	10.92	11.37	10.22	17.53	24.84	24.83	21.61	18.96
	S.D.	0.21	1.81	1.46	1.54	1.76	1.75	1.73	1.91	2.11
	Min.	13.09	8.71	9.23	8.20	14.85	21.77	22.09	18.95	16.32
	25%	13.15	9.72	10.44	9.14	16.57	23.92	23.98	20.60	17.60
	50%	13.23	10.27	11.15	9.80	17.14	24.87	24.58	21.38	18.33
	75%	13.42	12.43	12.32	11.40	18.90	25.68	25.50	21.97	20.19
	Max.	13.68	13.47	13.81	12.66	20.21	27.27	27.67	25.31	22.95

Table S1. contd.

Intermediate	Mean	23.30	15.92	15.85	14.79	16.63	20.38	21.58	20.88	19.80
	S.D.	2.67	2.06	1.93	1.98	2.74	3.21	3.15	3.12	2.99
	Min.	18.23	11.61	11.39	10.17	11.06	13.14	13.82	13.05	12.59
	25%	21.75	14.64	14.57	13.89	14.97	18.24	19.72	19.10	17.92
	50%	23.30	15.92	15.76	14.59	16.36	20.32	21.40	20.71	19.80
	75%	25.17	17.11	16.97	15.50	17.83	21.93	23.41	22.26	21.09
	Max.	29.94	20.83	19.87	18.26	22.11	26.43	27.21	26.46	25.17
Grey	Mean	18.59	11.34	11.02	10.27	10.69	12.53	12.89	12.31	12.66
	S.D.	5.03	4.93	4.95	4.54	4.82	5.84	5.88	5.38	4.61
	Min.	11.77	4.74	4.40	4.11	4.44	5.33	5.68	5.78	6.61
	25%	13.36	6.43	6.68	6.32	7.21	8.36	8.52	8.39	9.45
	50%	18.02	10.36	10.02	9.13	9.47	10.64	10.87	10.71	11.79
	75%	23.87	15.98	15.45	13.94	14.44	17.44	17.77	16.89	15.80
	Max.	25.66	18.32	17.97	17.94	19.74	23.46	24.54	24.01	23.80
Vegetation	Mean	5.10	5.6	6.62	6.90	8.09	20.23	13.99	9.51	20.60
	S.D.	2.15	2.35	3.15	3.51	3.03	5.21	4.21	3.06	4.32
	Min.	2.54	3.25	3.33	2.99	3.93	10.83	7.86	4.97	11.94
	25%	4.02	4.21	4.47	4.52	5.68	17.47	11.26	7.13	18.21
	50%	5.25	5.58	6.24	6.73	8.55	20.22	13.58	9.05	20.43
	75%	6.35	6.15	8.05	9.29	12.25	22.69	15.97	10.82	22.88
	Max.	10.64	13.54	14.30	15.10	16.82	30.40	23.50	18.63	30.12
