# Supplementary material for: Spectral Sensitivities and Color Signals in a Polymorphic Damselfly
Source: PLoS One. 2014 Jan 31;9(1):e87972. doi: 10.1371/journal.pone.0087972 (PMC3909319; doi:10.1371/journal.pone.0087972)
Supplement: Table S2 — One sample t-test of chromatic and achromatic contrasts under twilight and morning light compared with the jnd threshold value, 1. (PDF) [file pone.0087972.s003.pdf]

Supporting Information

Table S2. One sample t-test of chromatic and achromatic contrasts under twilight and morning light compared with the *jnd* threshold value, 1. Individual analyses were conducted on contrast data obtained for gender comparison (inter-sexual: males vs. female morphs), within gender (intra-sexual: female morph vs. female morph), and between individual morph and background vegetation.

	Twilight				Morning			
	Chromatic		Achromatic		Chromatic		Achromatic	
	$t_{(df)}$	P	$t_{(df)}$	P	$t_{(df)}$	P	$t_{(df)}$	P
Inter-sexual								
Male-Andromorph	42.35 (588)	<0.01	53.17 (588)	<0.01	30.41 (588)	<0.01	56.61 (588)	<0.01
Male-Green	85.25 (681)	<0.01	118.4 (681)	<0.01	91.27 (681)	<0.01	111.3 (681)	<0.01
Male-Intermediate	137.4 (1456)	<0.01	242.8 (1456)	<0.01	161.2 (1456)	<0.01	261.4 (1456)	<0.01
Male-Grey	74.25 (466)	<0.01	171.1 (466)	<0.01	73.94 (466)	<0.01	180.2 (466)	<0.01
Intra-sexual								
Green-Andromorph	57.64 (417)	<0.01	52.25 (417)	<0.01	65.52 (417)	<0.01	47.68 (417)	<0.01
Green-Intermediate	78.96 (1033)	<0.01	215.4 (1033)	<0.01	86.2 (1033)	<0.01	228.4 (1033)	<0.01
Green-Grey	22.89 (329)	<0.01	68.75 (329)	<0.01	29.81 (329)	<0.01	81.47 (329)	<0.01
Intermediate-Andromorph	137.1 (892)	<0.01	105.4 (892)	<0.01	142.8 (892)	<0.01	47.68 (892)	<0.01
Intermediate-Grey	19.56 (704)	<0.01	151.7 (704)	<0.01	24.45 (704)	<0.01	160.3 (704)	<0.01
Grey-Andromorph	47.58 (284)	<0.01	71.25 (284)	<0.01	49.96 (284)	<0.01	90.11 (284)	<0.01
Against vegetation								
Male-Vegetation	111.9 (1239)	<0.01	344.3 (1239)	<0.01	127.4 (1239)	<0.01	317.5 (1239)	<0.01
Andromorph-Vegetation	260.1 (759)	<0.01	179.4 (759)	<0.01	213.2 (759)	<0.01	163.1 (759)	<0.01
Green-Vegetation	42.77 (879)	0.11	78.48 (879)	<0.01	37.51 (879)	0.06	64.02 (879)	<0.01
Intermediate-Vegetation	222.1 (1879)	<0.01	261.1 (1879)	<0.01	248.2 (1879)	<0.01	154.4 (1879)	<0.01
Grey-Vegetation	83.33 (599)	<0.01	50.01 (599)	0.129	94.27 (599)	<0.01	72.14 (599)	<0.01
